# Orthoplastic management of distal tibia bone infection using Masquelet technique and PRECICE nail: A case report^☆^

**DOI:** 10.1016/j.tcr.2023.100834

**Published:** 2023-05-03

**Authors:** Mauricio Zuluaga, Sergio Cadavid, Federico Reina, Alma Reyes-Arceo, Fernando Benedetti

**Affiliations:** a Limb Lengthening and Reconstruction Unit, Clínica Imbanaco Grupo QuirónSalud, Cali, Colombia; b Faculty of Health Sciences, Pontificia Universidad Javeriana Cali, Colombia; c Orthopedic Surgery Residency Program, Fundación Universitaria de Ciencias de la Salud FUCS, Bogotá, Colombia

**Keywords:** Distal tibia fracture, Free flap, Soft tissue defect, Bone infection

## Abstract

The orthoplastic treatment of post-traumatic bone infections is complex and requires a multidisciplinary approach using both orthopedic and plastic surgery principles. Its primary goal is to achieve rapid control of the infection through aggressive debridement of the affected tissue, in order to perform a complete reconstruction of the limb. This allows both its salvage and restoration of function. We present a patient with septic non-union secondary to distal tibia fracture with a bone defect of 7 cm and severe soft tissue injury. The treatment was divided into three stages. First, the infection was controlled by radical debridement, limb shortening, and temporary stabilization. Second, early reconstruction was initiated utilizing the first stage of the Masquelet's induced membrane technique (MIMT), and soft tissue coverage with free flap. Third, MIMT was finalized, and bone lengthening with PRECICE nail was performed. We consider this approach effective as it can offer early recovery with optimal functional and aesthetic results in bone defects associated with coverage defects.

## Introduction

**Fig. 1:**
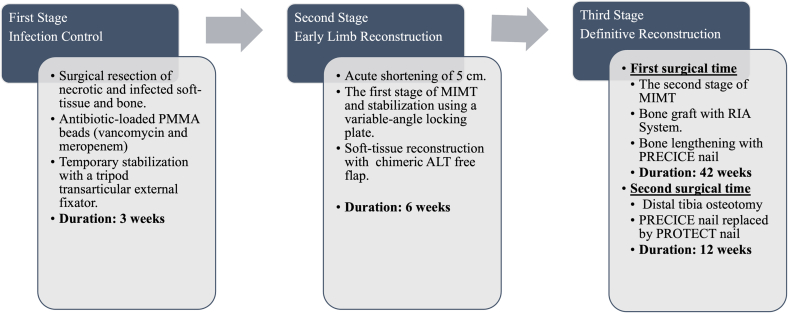
Description of the three-stage approach designed for this case for achieving limb salvage.

Surgical management of tibial fracture infection usually results in bone and soft tissue coverage defects during eradication; hence, different reconstruction options have been proposed. These include bone grafting, bone substitutes, Masquelet's induced membrane technique (MIMT), and distraction osteogenesis (e.g., Ilizarov Technique) [1]. Local or free microvascular flaps are an option for soft tissue defects [2]. Although each reconstruction option has its advantages and disadvantages, a combined approach of multiple surgical techniques to take advantage of the benefits of each is not common. In the following case, we discuss a patient with septic non-union secondary to distal tibia fracture with a bone defect of 7 cm and severe soft tissue injury, which was managed using an orthoplastic approach (Fig. 1).

## Case presentation

**Fig. 2:**
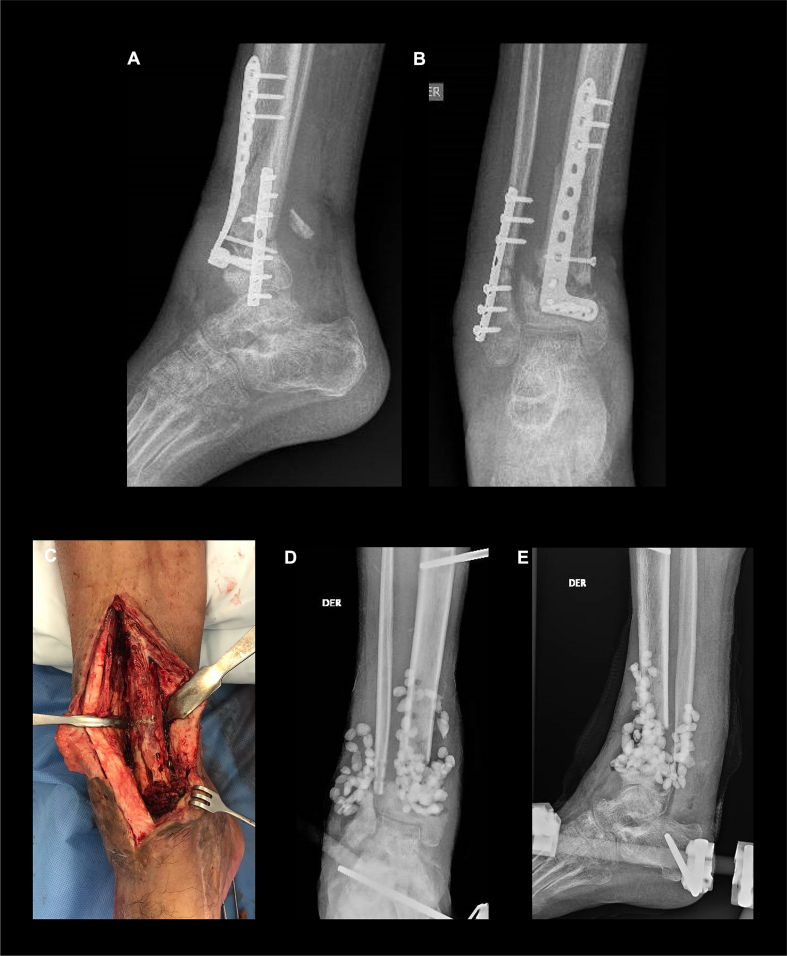
A–B): A 46-year-old man with a type IIIa open fracture in his right leg. X-rays show non-union in the distal tibia. C) X-ray showing infected soft tissue and bone. D–E) Stage 1 with resection of non-union focus and placement of antibiotic beads.

Following a high-energy trauma traffic accident, a 46-year-old man suffered a type IIIA open fracture of the distal tibia and fibula of his right leg, which was managed with open reduction and internal fixation at another institution. One week after osteosynthesis, he presented with associated material exposure that was treated with retention of osteosynthesis material, surgical debridement, application of negative pressure, and intravenous antibiotics. The patient had an unfavorable outcome leading to chronic infection, and transtibial amputation procedure was recommended. At 28 weeks post injury, the patient consulted our institution presenting with multiple persistent fistulas at the surgical site with purulent discharge, unstable scar, and loss of function associated with rigid equinus deformity and malrotation. X-rays showed atrophic non-union in the distal tibia and varus deformity with loosening of material (Fig. 2A–B).

The proposed management was divided into three stages: (1) eradication of the infection; (2) early bone and soft tissue reconstruction; (3) definitive skeletal reconstruction (Fig. 1). The first stage began 28 weeks post injury when the following procedures were performed: fistulectomy, resection of unstable scar and fibrosis, removal of osteosynthesis material, and radical debridement (soft tissues/non-union bone segment) (Fig. 2C). Intraoperative specimens were collected and a resulting bone defect of 7 cm in the distal tibia was quantified. Bone was then stabilized using a tripod transarticular external fixator, and polymethyl-methacrylate (PMMA) beads were placed with vancomycin 2 g and meropenem 1 g (Fig. 2D–E). A methicillin-sensitive *Staphylococcus aureus* was identified, which was treated with cefazolin. In a subsequent surgery, an evaluation of infection control was made and a negative pressure system with silver dressings was placed.

**Fig. 3:**
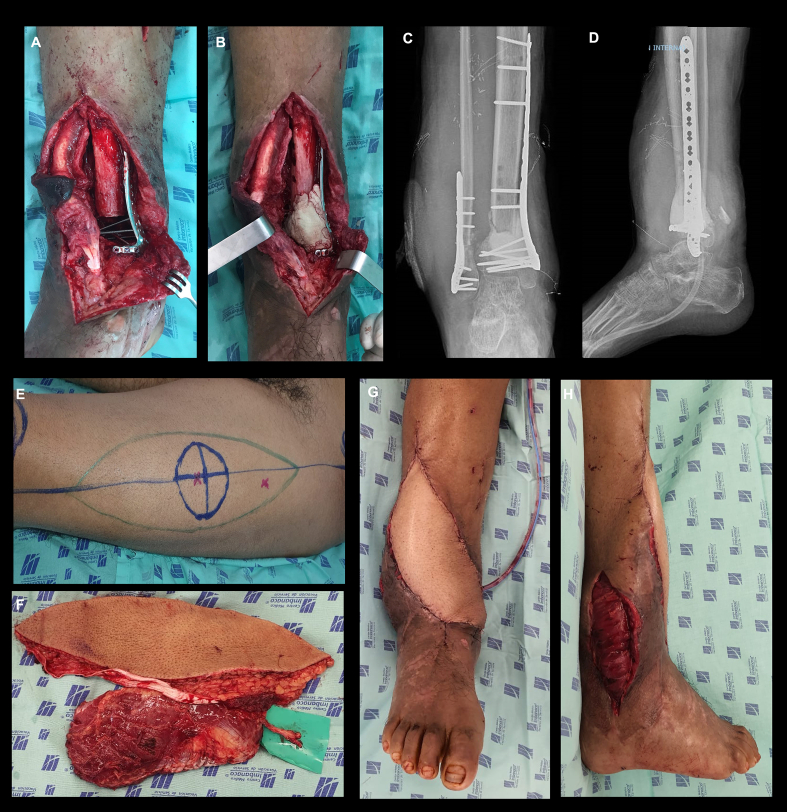
A–D) Osteosynthesis of distal tibia and fibula using a variable-angle locking plate, and the first stage of MIMT (Stage 2). E). Image shows the surgical planning of the anterolateral free flap. F) Image shows the Chimeric ALT free flap. G–H) Image after performing the reconstruction of the tissue defect with a free flap. (Stage 2).

Six days later (31 weeks post injury), the second stage using an orthoplastic approach began once satisfactory postoperative soft tissue was evidenced. Early skeletal reconstruction included acute shortening with fibula osteotomy (5 cm), osteosynthesis of both distal tibia and fibula segments with a variable angle locking plate, and the first stage of MIMT. A PMMA spacer of gentamicin plus vancomycin was used by filling the residual bone defect (2 cm) (Fig. 3A–D). A chimeric ALT free flap was performed in conjunction with the reconstructive microsurgery group (Fig. 3E–H). The patient had a favorable clinical result with an adequate flap integration process, wound healing, and no recurrence of infection. After this stage, the patient recovered joint function and began partial weight bearing.

**Fig. 4:**
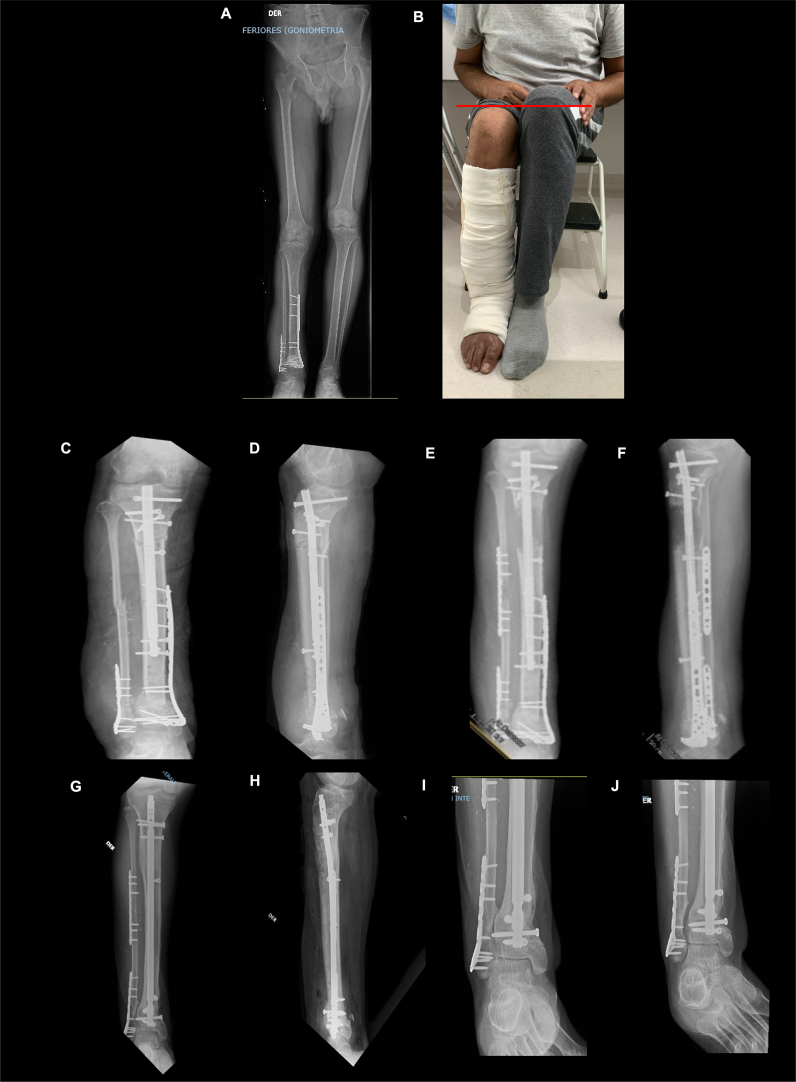
A) Orthoradiography with length discrepancy in lower limbs of 5 cm in relation to the contralateral limb secondary to acute shortening associated with 25 mm of bone defect in the tibial pilon. B) Red line highlights the discrepancy. C–D) X-ray Anteroposterior and lateral PRECICE nail placement with proximal metaphyseal osteotomy. E–F) Stabilization of the fibula with a locking compression plate. G–H): Consolidation of regenerated proximal metaphyseal. I–J): Consolidation of Masquelet distal tibia.

During the third stage (37 weeks post injury), limb shortening was managed through the placement of a PRECICE nail (NuVasive Specialized Orthopedics, San Diego, CA, USA) with percutaneous proximal metaphyseal osteotomy and diaphyseal osteotomy of the fibula for progressive bone lengthening (Fig. 4A–B). Additionally, the second stage of MIMT was performed for PMMA removal and placement of reamer-irrigator aspirator (RIA; Depuy Synthes; Johnson & Johnson Co. Inc., NJ, USA) autograft for the ipsilateral femur that was mixed with 3 × 0 cm^3^ of cancellous chips, achieving a complete filling of the defect (Fig. 4C–D). At forty-nine weeks post injury (3 months after the last surgery), the planned lengthening and equalization of the length discrepancy had been achieved, so the last stage of reconstruction was performed by stabilizing the fibula with a locking compression plate (LCP) (Fig. 4E–F). However, a residual valgus deformity was identified.

At 79 weeks post injury and 42 weeks after the PRECICE nail insertion, the nail was changed to an antibiotic-coated nail (TNS PROTECT®, DePuy Synthes) to avoid possible colonization and reactivation of infection. A varus osteotomy of the distal tibia was also performed because the patient had ankle pain when walking and some restrictions of activities of daily living.

**Fig. 5:**
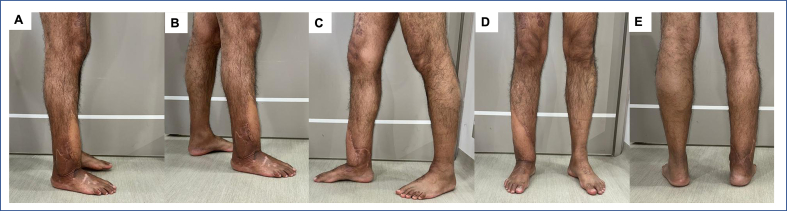
The photograph shows good aesthetic results after the surgery.

X-rays showed consolidation and regeneration of the proximal bone and consolidation of the bone defect in the distal tibia (Fig. 4G–J). Twelve weeks after the last surgery (91 weeks post injury), the patient returned to his work activities with no signs of infection and walked without external aids (Fig. 5). From the time the patient was admitted to our clinic to the time complete recovery was achieved, the duration of treatment was 63 weeks. The latest follow-up was performed 166 weeks post injury (∼3.2 postoperative years) and yielded the same favorable clinical outcomes, reporting an EQ-5D-3L profile of “21121” without severe disability in any of the 5 dimensions of the scale. The EQ-VAS score was 90 points.

## Discussion

The development of infection after surgical management of a fracture negatively affects clinical outcomes, requiring more complex procedures that worsen the prognosis. Furthermore, reconstruction is not always possible in critical bone defects with soft tissue coverage defects, and radical treatments such as amputation are warranted in severe cases. In these cases, the multidisciplinary orthoplastic approach offers comprehensive reconstructive management to achieve limb salvage, allowing the treatment of neurovascular lesions, bone reconstruction, management of soft tissue coverage defects, and early physical rehabilitation. Consequently, the orthoplastic approach improves the chances of success when compared to the traditional fragmented approach, with advantages such as fewer additional interventions, reduced postoperative pain, shorter hospital stays, lower risk of infection, and faster recovery of limb function [2], [3], [4].

This study describes our experience using an orthoplastic approach based on three stages in a patient with infected non-union of the tibial pilon. The first stage was planned to control the infection and the last two stages were to achieve the reconstruction of the bony defect and soft tissue coverage. In the skeletal reconstruction, a novel approach based on MIMT plus the use of autografts, PRECICE nail, and ALT flap was proposed. The treatment was completed with the conversion to TNS-PROTECT nail to increase the stability of the reconstruction and decrease the risk of reinfection.

In this case report, a salvage therapeutic strategy was designed based on the diamond concept proposed by Giannoudis for the treatment of a periarticular fracture of the distal tibia [5]. Thus, the entire defect was not initially shortened to avoid the risk of vascular complications, and instead, MIMT was used, which has shown good results in the consolidation of bone defects of 2 to 5 cm [6]. The use of the ALT flap allowed coverage of the reconstruction, serving as a barrier, and providing vascular support, guaranteeing one of the points of the diamond concept to achieve bone consolidation [5]. In addition, the PRECICE nail was preferred for proximal lengthening over external fixation to reduce the psychological impact and allow earlier rehabilitation with fewer adverse effects in order to benefit the consolidation of the segment as suggested in the literature [7].

To the best of our knowledge, this is the first report describing an orthoplastic approach combining MIMT and PRECICE nail in the management of septic non-union of tibial pilon fractures. Other proposals have combined the orthoplastic approach with definitive bone reconstruction using principles of distraction osteogenesis with external fixation, such as Corona et al., who reported 31 patients with a limb salvage rate of 93.5 % (29/31) and return to work rate of 83 % [8]. Similarly, Bauer et al., in a cohort of 50 patients reported that in 95 % of the cases the limb was not amputated [9].

## Conclusion

Based on our experience, we believe that the orthoplastic approach with MIMT, PRECICE nail, and ALT free flap for management of massive bone defects of the tibial pilon with residual limb shortening and soft tissue coverage defect, can provide faster recovery and improved restoral of function with good aesthetic results.

## Declaration of competing interest

The authors have no conflict of interest to declare.
